# Supplementary material for: Synthesis and Characterization of MnIn2S4/Single-Walled Carbon Nanotube Composites as an Anode Material for Lithium-Ion Batteries
Source: Nanomaterials (Basel). 2024 Apr 19;14(8):716. doi: 10.3390/nano14080716 (PMC11053989; doi:10.3390/nano14080716)
Supplement: Supplementary file 1 [file nanomaterials-14-00716-s001.zip › nanomaterials-2908566-supplementary.pdf]

Supporting Information

Synthesis and characterization of $MnIn_2S_4$/SWCNT composites as an anode material for Lithium-ion batteries

Pei-Jun Wu,[1] Chia-Hung Huang,[2,3] Chien-Te Hsieh[4,5*] and Wei-Ren Liu[1,*]

[1]*Department of Chemical Engineering, Chung Yuan Christian University, R&D Center for Membrane Technology, Center for Circular Economy 200 Chung Pei Road, Chungli District, Taoyuan City, 32023, Taiwan, R.O.C.*

[2]*Department of Electrical Engineering, National University of Tainan, No. 33, Sec. 2, Shulin St., West Central District, Tainan City 700, Taiwan; chiahung@mail.mirdc.org.tw*

[3]*Metal Industries Research and Development Centre, Kaohsiung 701, Taiwan*

[4]*Department of Chemical Engineering and Materials Science, Yuan Ze University, Taoyuan 32003, Taiwan; cthsieh@saturn.yzu.edu.tw (C.T.H)*

[5]*Department of Mechanical, Aerospace, and Biomedical Engineering, University of Tennessee, Knoxville, TN 37996, United States; cthsieh@saturn.yzu.edu.tw (C.T.H)*

* *Correspondence: wrliu1203@gmail.com ; cthsieh@saturn.yzu.edu.tw ; Tel.: +886-3-265-4140; fax:+886 3-265-4199*

**E-mail address: WRLiu1203@gmail.com*

Tel:+886 3-265-4140; fax:+886 3-265-4199

Table S1. Grain size of pristine MIS and as-synthesis MIS/SWCNTs.

Samples	Grain Size (Å)
MIS	159
MIS/SWCNTs	103

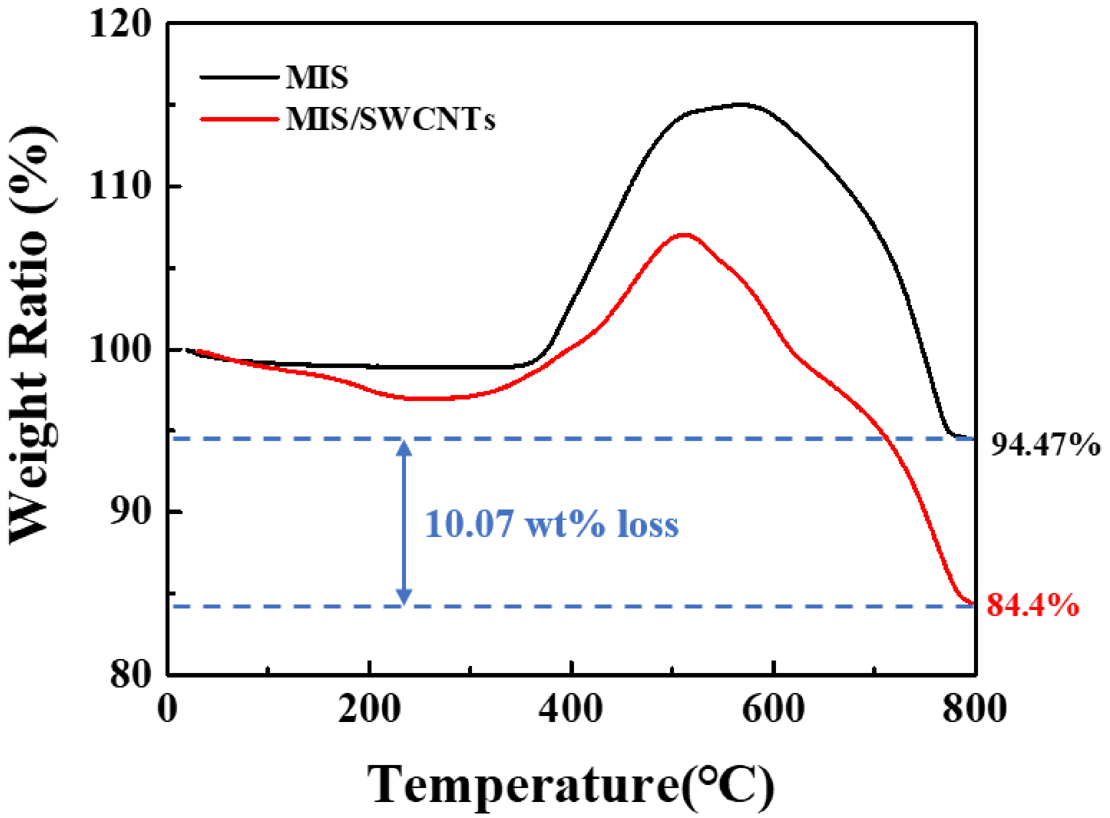

Figure S1. TGA of MIS and MIS/SWCNTs at heating rate of 10°C/min in air atmosphere.

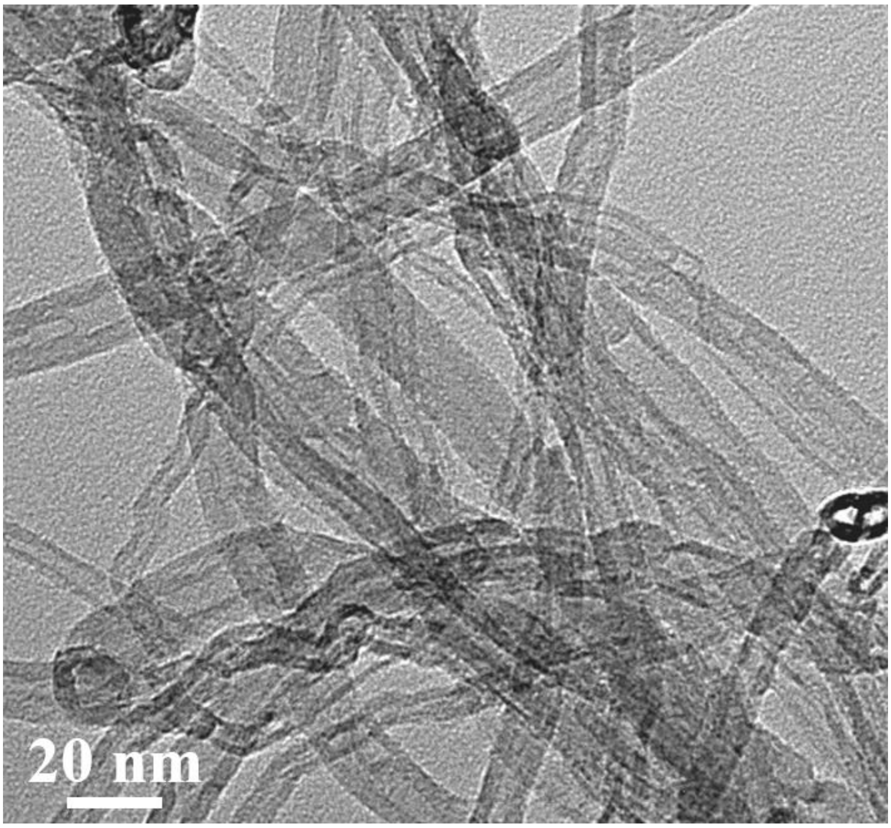

Figure S2. HRTEM of image of SWCNTs.

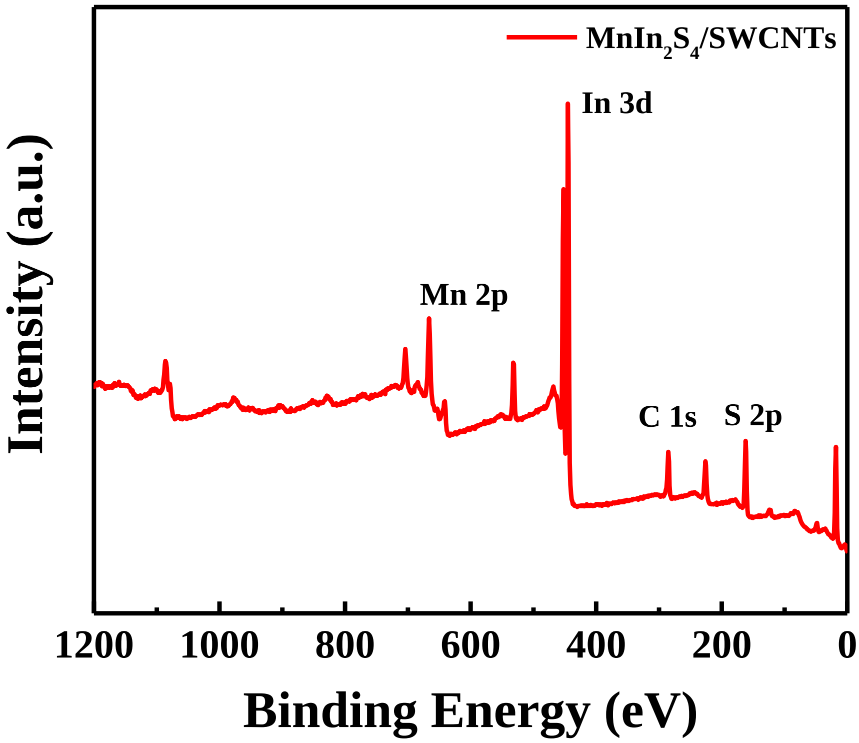

Figure S3. XPS survey spectrum of MIS/SWCNTs with high resolution.

Table S2. Electrochemical impedance parameters estimated from equivalent circuit.

Samples	R_s (Ω)	R_{SEI} (Ω)	R_{CT} (Ω)	Slope	R^2	D_{Li^+} (cm^2/s)
MIS	3.78	106.3	72.96	14.57	0.890	1.7 x10^{-14}
MIS/SWCNTs	14.83	60.2	27.93	4.63	0.927	1.7 x10^{-13}

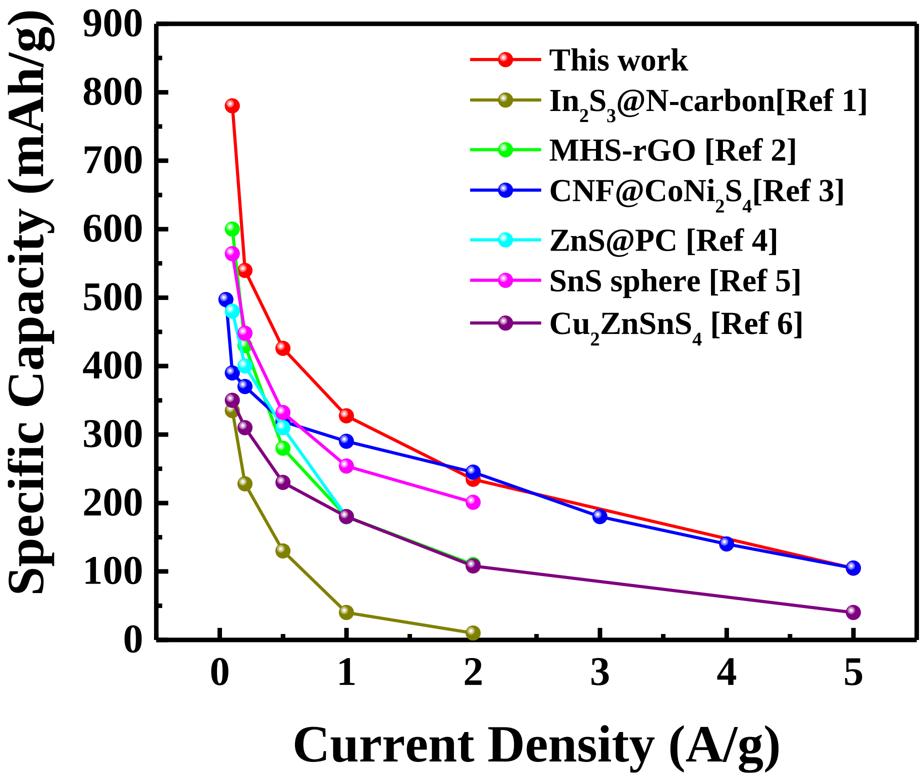

Figure S4. Rate performance comparison of as-earlier studies of transition metal sulfide anode for LIBs.

Table S3. Comparison of electrochemical performance with reported transition metal sulfide as anode materials for Lithium-ion batteries.

Samples	*ICE	Capacity (mAh/g)	After nth cycles	Current density (A/g)	Ref.
MIS/SWCNTs	61%	536	100	0.2	This work
In_2S_3@N-carbon	44%	485	200	0.1	[1]
MHS-rGOs	69%	500	50	0.2	[2]
CNF@$CoNi_2S_4$	n/a	~520	100	0.1	[3]
ZnS@PC	n/a	438	300	0.1	[4]
SnS sphere	64%	414	100	0.1	[5]
Cu_2ZnSnS_4	34%	234	30	0.1	[6]

References

[1] L. Sun, X. Liu, T. Ma, L. Zheng, Y. Xu, X. Guo, J. Zhang, "In_2S_3 nanosheets anchored on N-doped carbon fibers for improved lithium storage performances," *Solid State Ionics*, 329 (2019), 8-14

[2] D. Chen, H. Quan, G.S. Wang, L. Guo, "Hollow α-MnS spheres and their hybrids with reduced graphene oxide: synthesis, microwave absorption, and lithium storage properties," *Chempluschem*, *78(8)*(2013), 843-851.

[3] A. Jagadale, X. Zhou, D. Blaisdell, S. Yang, "Carbon nanofibers (CNFs) supported cobalt- nickel sulfide ($CoNi_2S_4$) nanoparticles hybrid anode for high performance lithium ion capacitor," *Scientific Reports*, *8*(2018), 1602..

[4] Y. Fu, Z. Zhang, X. Yang, Y. Gan, W. Chen, "ZnS nanoparticles embedded in

porous carbon matrices as anode materials for lithium ion batteries," *RSC Advances*, *5(16)*(2015), 86941-86944.

[5] J. Ren, R.P. Ren, Y.K. Lv, "Hollow spheres constructed by ultrathin SnS sheets for enhanced lithium storage," *Journal of Materials Science*, 55(17)(2020), 7492-7501.

[6] X. Yang, J. Xu, L. Xi, Y. Yao, Q. Yang, C. Chung, C. Lee, "Microwave-assisted synthesis of Cu_2ZnSnS_4 nanocrystals as a novel anode material for lithium ion battery," *Journal of Nanoparticle Research*, *14(6)*(2012), 931.
